# Stress-induced sleep-like inactivity modulates stress susceptibility in mice

**DOI:** 10.1038/s41598-020-76717-8

**Published:** 2020-11-13

**Authors:** Midori Nagai, Hirotaka Nagai, Chisato Numa, Tomoyuki Furuyashiki

**Affiliations:** 1 Division of Pharmacology, Graduate School of Medicine, Kobe University, 7-5-1 Kusunoki-cho, Chuo-ku, Kobe, 650-0017 Japan; 2 Japan Agency for Medical Research and Development, Tokyo, 100-0004 Japan

**Keywords:** Sleep, Sleep deprivation, Wakefulness, Neuroscience, Learning and memory, Social behaviour, Stress and resilience, Depression

## Abstract

Severe environmental and social stress induces dysregulation of sleep along with mood and cognitive disturbances. However, the role and mechanism of this sleep dysregulation remain elusive. Here we evaluated sleep-like inactivity measured by voluntary movements and its relationship to social behaviors in mice without or with social defeat stress as well as the stressed mice with subsequent sleep deprivation. Social defeat stress immediately induced sleep-like inactivity with decreased body temperature. In the social interaction test, the control mice showed high social interest and its correlation with social sniffing intensity, the latter of which indicates positive valence of social sniffing. After the stress, these social characteristics were maintained in stress-resilient mice, but disrupted in stress-susceptible mice, leading to social avoidance. Sleep deprivation after the stress decreased social sniffing intensity along with reduced social interest, but enhanced the exploratory activity with the positive valence of social sniffing. We also found by c-Fos immunohistochemistry that the stress activated sleep-related brain regions, the dorsomedial hypothalamus and ventrolateral periaqueductal gray. Collectively, these findings show that stress activates sleep-related brain regions and induces sleep-like inactivity, contributing to multiple roles of stress-induced sleep for social behaviors.

## Introduction

Severe environmental and social stress disturbs mental functions and predisposes to mood disorders in humans^1^. Chronic stress in rodents, such as chronic mild stress and repeated social defeat stress, causes depressive-like behaviors and has been used to study the pathology of mood disorders^2–4^. Sleep disturbances are major clinical issues in mood disorders^5^. Both insomnia and hypersomnia have been associated with major depressive disorder, and these sleep disturbances are thought to affect the relapse and recurrence of this disorder^6^. Rodent studies have also analyzed the effects of stress on sleep/wake regulation and found that either acute or chronic stress increases sleep time during the dark phase^7–12^. However, the role of sleep disturbances in stress and depression remains elusive.

Neural circuits that underlie sleep/wake regulations have been studied in animal models. These studies have identified sleep-promoting brain regions and wake-promoting brain regions, which exert mutual inhibitions through reciprocal connections^13,14^. Thus, sleep-promoting brain regions, such as the ventrolateral preoptic area (VLPO) and median preoptic area (MnPO), send inhibitory projections to wake-promoting brain regions including monoaminergic, cholinergic and histaminergic neurons. Neurons in wake-promoting neurons send their projections to and inhibit sleep-promoting brain regions. These reciprocal connections regulate the balance between the activities of sleep-promoting and wake-promoting brain regions, thereby shaping the pattern of sleep/wake cycles^14^. In addition, other brain regions, such as the dorsomedial hypothalamus (DMH) and ventrolateral periaqueductal gray (VLPAG), are involved in regulating sleep state^15,16^. For example, a distinct subset of DMH neurons suppresses rapid eye movement (REM) sleep and promotes non-REM sleep, whereas another subset of neurons exerts the opposite effects. The activation of VLPAG neurons has been shown to gate REM sleep. However, whether these brain regions related to sleep and wake are affected by stress has not been examined.

In this study, we evaluated sleep-like inactivity measured by voluntary movements and its relationship to social behaviors in three groups of mice: those without or with social defeat stress as well as the stressed mice with subsequent sleep deprivation. We also examined neuronal activity in brain regions related to sleep and wake regulations by c-Fos immunohistochemistry without or with social defeat stress. Our findings suggest multiple roles of stress-induced sleep-like inactivity for social behaviors and its relationship to the activation of sleep-related brain regions.

## Methods

### Animals

Adult male C57BL/6N mice of 7–10 weeks old and male ICR mice retired from breeding were purchased from Japan SLC (Shizuoka, Japan). Mice were housed in groups of 4–5 mice per cage and maintained in the animal facility with constant temperature and humidity on a 12 h light/12 h dark cycle with food and water available ad libitum for at least one week before the experiments. Male C57BL/6N mice used in behavioral experiments were divided into three groups. In the “Control” group, the mice were transferred to a novel cage without an aggressor ICR mouse for 10 min (“cage transfer”). In the “Stress” group, the mice received social defeat stress from an aggressor ICR mouse for 10 min instead. In the “Stress + Sleep dep” group, the mice received the stress for 10 min immediately followed by sleep deprivation for 6 h. Male C57BL/6N mice used for immunohistochemistry were divided into the “Control” and “Stress” groups, each of which was treated similarly to the behavioral experiments. All procedures for animal care and use were in accordance with the National Institutes of Health Guide for the Care and Use of Laboratory Animals and were approved by the Animal Care and Use Committees of Kobe University Graduate School of Medicine.

### Measurement of voluntary movement and core body temperature

Voluntary movement and core body temperature were recorded as previously described^17^ with minor modifications. Briefly, under isoflurane anesthesia, male C57BL/6N mice of 7–10 weeks were implanted with a small wireless sensor (Nano tag, Kissei Comtec, Matsumoto, Japan; https://www.sleepsign.com/nanotag/spec.html) with its size of 18.8 mm × 14.2 mm × 7.1 mm and its weight of 2.7 g into their abdominal cavity. After surgery, the mice were singly housed for post-operation recovery for at least 1 week. During the measurement, we detected voluntary movement every 12 s and the body temperature every minute. To measure voluntary movement, this sensor recorded three-dimensional acceleration vectors at 25 Hz and counted the frequency that the acceleration vector-synthesized waveform exceeded a given threshold. We defined inactive periods if voluntary movements were absent in two consecutive time points, and defined other periods as active periods. The proportion of inactive periods (i.e. the number of inactive time points divided by the total number of measurements) in every 1-h bin was plotted in percentage and analyzed. This definition is derived from previous studies in which the continuous lack of voluntary movements during 30–40 s was defined as sleep-like inactivity^18–20^. This definition has been shown to yield a consistent estimate of total sleep time with 90% accuracy based on EEG recording^18,20^. The temperature was recorded inside the sensor with the maximum error of 0.5 °C.

### Schedule of behavioral experiments

**Figure 1:**
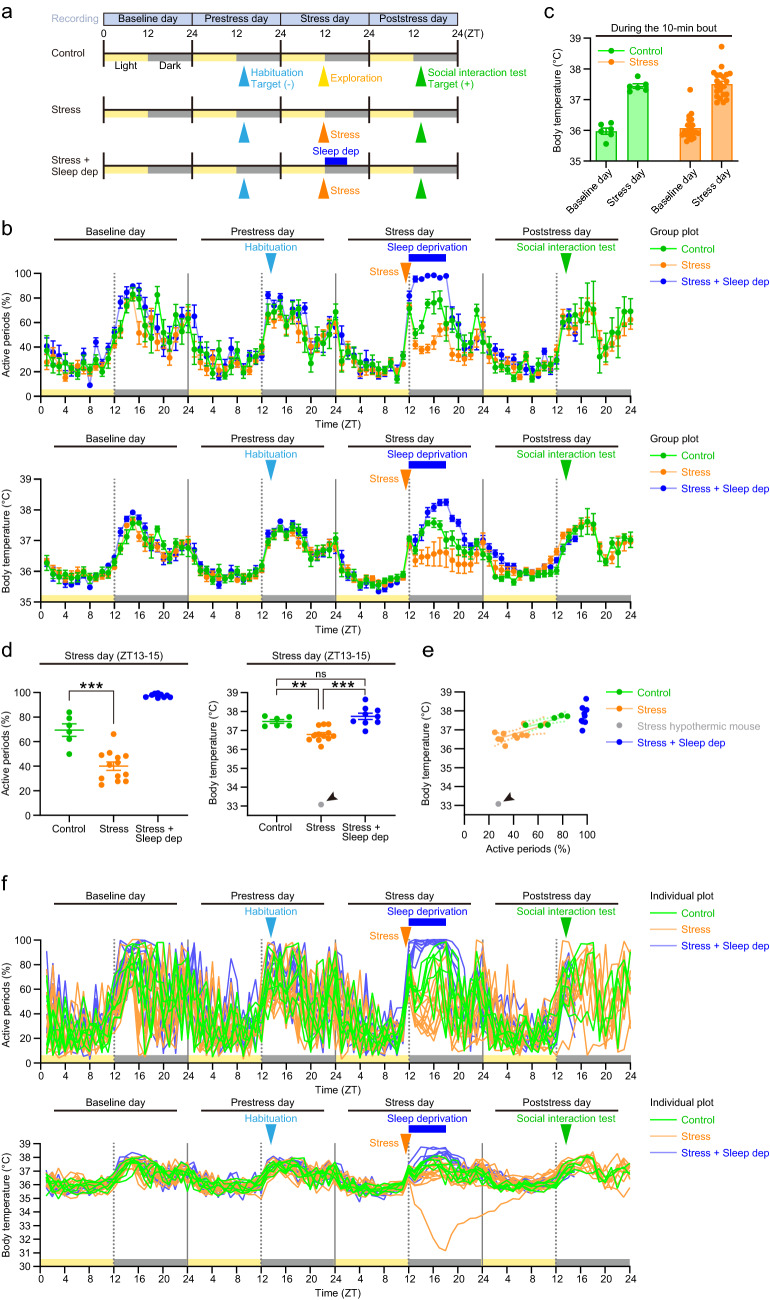
Social defeat stress induces sleep-like inactivity with decreased body temperature. (**a**) A behavioral schedule. On the baseline day, mice implanted with a small acceleration and temperature sensor in their abdominal cavity were left undisturbed in their home cages. On the prestress day, they received habituation to the behavioral chamber used in the social interaction test without an ICR target mouse (“Target (−)”) at ZT 13–15. On the stress day, they received social defeat stress for 10 min for stressed mice without (N = 13) or with (N = 9) sleep deprivation for 6 h (ZT 12–18) (“Stress” or “Stress + Sleep dep”, respectively), or cage transfer for 10 min (N = 6, “Control”) just prior to ZT 12. On the poststress day, they received the social interaction test with an ICR target mouse (“Target (+)”) at ZT 13–15. The values during ZT 16–24 on the poststress day are missing in 3 control mice, 3 stressed mice and all stressed mice with sleep deprivation. (**b**) Time course plots of the proportion of active periods (above) and the core body temperature in each one-hour bin (below). (**c**) The core body temperature during either cage transfer (“Control”) or social defeat stress (“Stress”) on the Stress day and during the same ZT on the Baseline day. Note that the data with the stress are combined from both the “Stress” and “Stress + Sleep dep” groups, as the two groups were treated the same before sleep deprivation. (**d**) Mean values of the proportion of active periods (left) and the core body temperature (right) during ZT 13–15. Note that the arrowhead indicates one of the stressed mice with severe hypothermia, which was excluded from statistical analyses for the body temperature. (**e**) The relationship between the proportion of active periods and core body temperature during ZT 13–15. The solid and dotted lines indicate linear regression lines and their 95% confidence intervals for the respective behavioral conditions. One of the stressed mice with severe hypothermia was excluded from linear regression analysis for the stressed mice. (**f**) Time course plots of the proportion of active periods (above) and the core body temperature (below) in each one-hour bin for individual mice shown in (**b**). Values are expressed as means ± SEM. **P < 0.01; ***P < 0.001; ns, not significant for Tukey’s multiple comparisons test following one-way ANOVA.

After implanted with a small wireless sensor for voluntary movement and body temperature, male C57BL/6N mice were singly housed and subjected to behavioral experiments as follows (Fig. 1a). On the first day (baseline day), the measurement of the voluntary movement and body temperature started at the beginning of the light period, and the mice were left undisturbed in their home cages. On the second day (prestress day), they received habituation to the behavioral chamber used in the social interaction test without an ICR target mouse at zeitgeber time (ZT) 13–15. On the third day (stress day), they were subjected to cage transfer (“Control”) or received social defeat stress for 10 min during ZT 11–12, just prior to the dark phase, without or with subsequent sleep deprivation for 6 h during ZT 12–18 (“Stress” or “Stress + Sleep dep”, respectively). On the fourth day (poststress day), they received the social interaction test with an ICR target mouse at ZT 13–15. The measurement of the voluntary movement and body temperature ended at the end of the poststress day, except a subset of mice in which it was terminated right after the social interaction test (see the legend of Fig. 1a).

### Social defeat stress

Social defeat stress was performed as previously described with minor modifications^3,21–24^. To obtain aggressor ICR mice, male ICR mice were screened before social defeat stress based on their aggressiveness to a novel male C57BL/6N mouse for 3 min daily for 3 days. We assessed the aggressiveness by the latency and frequency of attacks during the observation period, and only used the ICR mice showing stable aggression for further experiments. Male C57BL/6N mice were transferred to the home cage of a male aggressor ICR mouse for 10 min during ZT 11–12. Control mice were treated the same as stressed mice, in that they were transferred to a novel cage without an ICR aggressor mouse, during the same ZT. Although all the mice were singly housed throughout for the wireless recording of voluntary movements and body temperature, the lack of social interaction does not induce apparent behavioral changes at least within three weeks (e.g., Nie et al.^22^, Niwa et al.^25^). After returned to their home cages, a subset of stressed mice was deprived of sleep as described later, whereas the remaining mice were left undisturbed.

### Sleep deprivation

Sleep deprivation was performed as previously described^19^. The combination of exposure to novel objects and gentle handling was used to perform sleep deprivation physiologically and effectively. Male C57BL/6N mice in the “Stress + Sleep dep” group were exposed to novel objects such as toys and plastics of different shape, color and texture to enforce wakefulness and were also poked with cotton swabs gently for 6 h after the stress, while singly housed at their home cages. Novel objects were frequently changed during sleep deprivation to facilitate the exploration of the objects. We only poked mice only when they showed drowsiness. Male C57BL/6N mice in the “Control” and “Stress” groups were also singly housed at their home cages, but were left undisturbed without either the exposure to novel objects or gentle handling.

### Social interaction test

The social interaction test following social defeat stress was performed as previously described^3^. We used a behavioral chamber with the size of 30 cm × 40 cm and with a metal meshwork placed at one end of the chamber. On the second day of the experiments (prestress day), at ZT 13–15, male C57BL/6N mice were habituated to the behavioral chamber by exploring it for 150 s. On the fourth day of the experiments (poststress day), the mice received the social interaction test at ZT 13–15. In this test, the mice were again allowed to freely explore the same behavioral chamber but with a novel ICR male mouse in the metal meshwork. Note that this ICR mouse was different from those used for social defeat stress. During the habituation and social interaction test, mouse behaviors were video-recorded and automatically analyzed by the SMART video tracking software (PanLab Harvard Apparatus, Holliston, MA, USA). Social interaction to an ICR mouse in the metal meshwork was evaluated by the time spent in the interaction zone and the avoidance zone, which are the areas at one side of the behavioral chamber with the metal meshwork and the opposite side, respectively.

### Detection of social sniffing

To quantify the level of social investigation during the social interaction test, we manually counted the time of social sniffing of a male C57BL/6N mouse to an ICR target mouse. To evaluate sniffing bouts with the length of seconds, we employed DeepLabCut, a deep learning-based algorithm which can accurately track respective body parts of a mouse during the social interaction test^26^. First, we extracted several images from the video data of respective mice and manually designate body parts to make training data. After the model was trained by 50,000 iterations, we performed the inference of pertinent body parts in the video data and confirmed its accuracy by manual inspection. A sniffing bout was detected when the nose of an experimental mouse stayed within 2 cm of the metal meshwork enclosing an ICR target mouse for 500 ms or more.

### Immunohistochemistry

Immunohistochemistry for c-Fos was performed as previously described^27^. It has been shown that neuronal activation induces transient expression of c-Fos protein at the peak of 90 min^28^. At 90 min after cage transfer (“Control”) or social defeat stress (“Stress”), male C57BL/6N mice were anesthetized with intraperitoneal injection of sodium pentobarbital (100 mg/kg, Nacalai Tesque, Kyoto Japan) and transcardially perfused with a flush of saline followed by 0.1 M sodium phosphate buffer containing 4% paraformaldehyde (Merck, Darmstadt, Germany). Brains removed from the mice were post-fixed in the same fixative at 4 °C overnight. After cryoprotection in Dulbecco’s modified phosphate buffered saline (D-PBS) containing 30% sucrose, the brains were frozen in OCT compound and cut into sections of 30-μm thickness using a cryostat (CM1860, Leica Biosystems, Wetzlar, Germany). The brain sections were incubated in blocking solution (D-PBS containing 1% normal donkey serum (017-000-121, Jackson ImmunoResearch Laboratories, Inc, West Grove, PA, USA) and 0.3% Triton X-100) for 1 h at room temperature (RT), followed by the incubation in the blocking solution with primary antibodies for c-Fos raised in rabbits (1:500 dilution; sc-52; Santa Cruz Biotechnology, Santa Cruz, CA, USA) at 4 °C for 2 days. After three times wash with D-PBS containing 0.3% Trixon-X 100 at RT, the sections were then incubated with Alexa Fluor 555-conjugated anti-rabbit IgG antibodies (1:1000 dilution; A31572; Thermo Fisher Scientific, Waltham, MA, USA) at 4 °C for 1 day. After three times wash with D-PBS containing 0.3% Triton X-100 at RT followed by a rinse with D-PBS, the sections were incubated with Hoechst 33,342 (Thermo Fisher Scientific; 2 μg/mL) in D-PBS at RT for 15 min. After twice wash with D-PBS at RT, the sections were dried on APS-coated glass slides (Matsunami Glass, Kishiwada, Japan) and mounted using a coverslip with the ProLong Gold antifadant (Thermo Fisher Scientific). Fluorescent images were taken with BZ-X710 (Keyence, Osaka, Japan).

To analyze the number of c-Fos-expressing cells, we defined brain areas based on the Allen Mouse Brain Atlas and a mouse brain atlas by Paxinos and Franklin^29^, as previously described^19,27^. We analyzed the ventrolateral preoptic area (VLPO), median preoptic nucleus (MnPO), medial septum (MS), lateral hypothalamus (LH), laterodorsal tegmental nucleus (LDT), dorsomedial hypothalamus (DMH), and ventrolateral periaqueductal gray (VLPAG). We applied the Transfluor application module of the Metamorph software (Molecular Devices Corporation, PA, USA) to detect and count objects that are within the range of 9–30 μm in diameter and brighter than a threshold determined by adjacent background signals. These objects were defined as c-Fos-expressing cells. We averaged the numbers of c-Fos-expressing cells in the same brain regions of two hemispheres in each mouse.

### Statistical analyses

**Figure 2:**
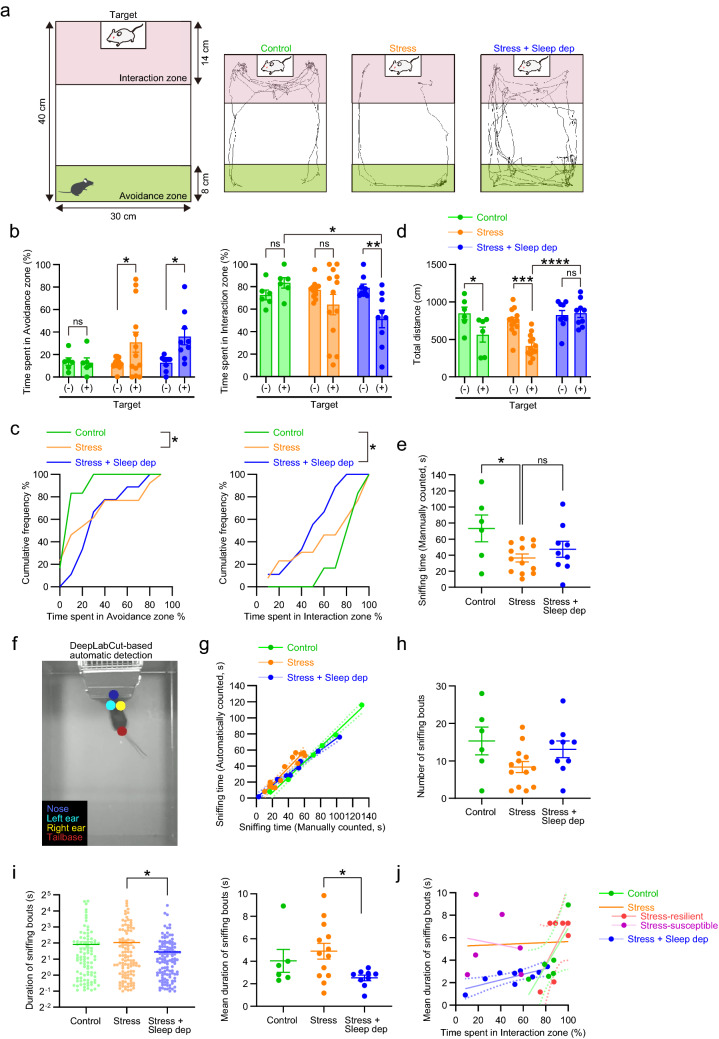
Stress-induced sleep-like inactivity is involved in multiple aspects of social behaviors. (**a**) A schematic of the social interaction test (left) and representative traces of behaviors of control mice (“Control”) and stressed mice without (“Stress”) or with sleep deprivation (“Stress + Sleep dep”) (right). (**b**) The proportion of the time spent in the avoidance zone (left) and the interaction zone (right) during the habituation without an ICR target mouse (−) or the social interaction test with an ICR target mouse (+). (**c**) The cumulative frequency of the proportion of the time spent in the avoidance (left) or interaction (right) zone shown in (**b**). (**d**) Total distance traveled during the habituation (−) or the social interaction test (+). (**e**) Total time of social sniffing to an ICR target mouse during the social interaction test by manual counting. (**f**) A representative image of the experimental mouse with DeepLabCut-based labeling of respective body parts, enabling automatic detection of social sniffing. (**g**) The relationship between total time of social sniffing with manual and automatic counting. The solid and dotted lines indicate linear regression lines and their 95% confidence intervals. (**h**) The number of sniffing bouts. (**i**) The duration of sniffing bouts (left) and its mean for individual mice (right). (**j**) The relationship between the time spent in the interaction zone and the mean duration of sniffing bouts. Each dot represents the values from an individual mouse. Stressed mice (without sleep deprivation) were analyzed as a whole or separately for stress-susceptible mice and stress-resilient mice. The solid and dotted lines indicate linear regression lines and their 95% confidence intervals (only for control mice, stress-resilient mice and stressed mice with sleep deprivation). Values are expressed as means ± SEM. *P < 0.05; **P < 0.01; ***P < 0.001; ****P < 0.0001; ns, not significant for Sidak’s multiple comparisons test following two-way repeated-measures ANOVA in (**b**, **d**), for Kolmogorov–Smirnov test in (**c**), and for Tukey’s multiple comparisons test following one-way ANOVA in (**e**, **i**).

**Figure 3:**
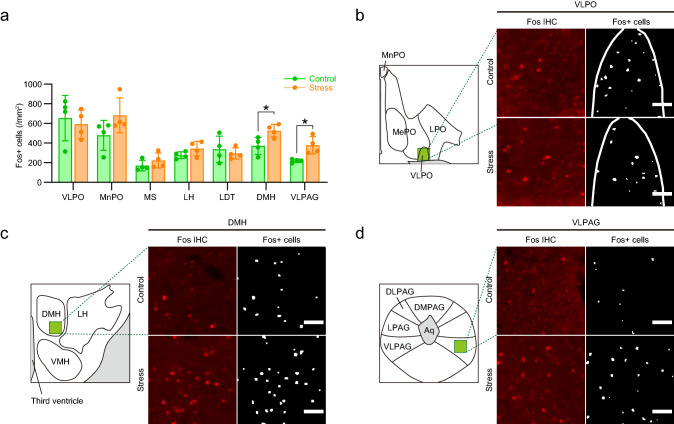
Stress increases c-Fos expression in sleep-related brain regions without affecting VLPO and MnPO. (**a**) The number of c-Fos expressing cells in each brain region normalized by area. Mice were sacrificed 90 min after cage transfer (“Control”) or social defeat stress (“Stress”) for 10 min. N = 4 for both control and stressed mice. (**b**–**d**) Representative images of c-Fos immunohistochemistry (“Fos IHC”) in the VLPO (**b**), DMH (**c**) and VLPAG (**d**). The cells expressing c-Fos were determined automatically and shown as Fos + cells. Scale bars: 50 μm. Values are expressed as means ± SEM. *P < 0.05 for unpaired *t* test in (**a**). *Aq* cerebral aqueduct, *DLPAG* dorsolateral periaqueductal gray, *DMH* dorsomedial hypothalamus, *DMPAG* dorsomedial periaqueductal gray, *LDT* laterodorsal tegmental nucleus, *LPAG* lateral periaqueductal gray, *LH* lateral hypothalamus, *LPO* lateral preoptic area, *MePO* medial preoptic area, *MnPO* median preoptic nucleus, *MS* medial septum, *VLPO* ventrolateral preoptic area, *VLPAG* ventrolateral periaqueductal gray, *VMH* ventromedial hypothalamus.

Data are expressed as means ± SEM, except for Supplementary Fig. S1, in which means ± SD are shown. Unpaired *t* test was used to compare the numbers of c-Fos-positive cells between the “Control” and “Stress” groups (Fig. 3a). Kolmogorov–Smirnov test was used to compare cumulative distributions of the time spent in the avoidance or interaction zone between the “Control”, “Stress”, and “Stress + Sleep dep” groups (Fig. 2c). One-way ANOVA followed by Tukey’s multiple comparisons test was used to compare the proportion of active periods (Fig. 1d), body temperature (Fig. 1d), manually counted sniffing time (Fig. 2e), or duration of sniffing bouts (Fig. 2i) among the “Control”, “Stress”, and “Stress + Sleep dep” groups. Two-way repeated measures ANOVA followed by Sidak’s multiple comparisons test was used to compare the time spent in the avoidance or interaction zone (Fig. 2b) and the total moved distance during the habituation and the social interaction test (Fig. 2d) among the “Control”, “Stress” and “Stress + Sleep dep” groups as a between-subjects factor, and between the absence and presence of an ICR target mouse as a within-subject factor. Linear regression and Pearson test were used to analyze correlations between the proportion of active periods and body temperature (Fig. 1e), between manually and automatically counted sniffing times (Fig. 2g), and between the time spent in the interaction zone and the mean duration of sniffing bouts (Fig. 2j). Analysis of covariance (ANCOVA) was used to compare the slopes and intercepts for the correlations of the proportion of active periods to body temperature between the “Control” and “Stress” groups, and the correlations of the time spent in the interaction zone to the mean duration of sniffing bouts between the high-social-interest mice from the “Control” and “Stress” groups and stress-susceptible mice from the “Stress” group. The analyses were performed with Prism 8.4 software (GraphPad Software, San Diego, CA, USA). *P* values less than 0.05 were considered significant. We included all the data for the analyses without any exclusion.

## Results

### Social defeat stress immediately induces sleep-like inactivity

As a baseline, all the mice showed clear nocturnal behavior with high activity during the dark phase and low activity during the light phase (Fig. 1a,b), consistent with previous literature^18,19^. Although body temperature was increased during social defeat stress or cage transfer (Fig. 1c), only social defeat stress showed long-lasting decrease in active periods and core body temperature for at least 3 h, relative to the control mice (Fig. 1b). The proportion of active periods was significantly decreased during ZT 13–15, from one to three hours after the stress (Fig. 1d; 69.4 ± 5.03% and 40.1 ± 3.36% for control and stressed mice, respectively; *P* < 0.0001 for Tukey multiple comparisons test). Since one stressed mouse showed severe hypothermia unlike the other stressed mice, we first excluded this mouse from the analyses of body temperature. The body temperature during the same period was decreased within the physiological range (37.47 ± 0.103 °C and 36.79 ± 0.106 °C for control mice and stressed mice, respectively; *P* = 0.0057 for Tukey multiple comparisons test). The body temperature was correlated to the proportion of active periods in both control and stressed mice (Fig. 1e; Pearson r = 0.8543, *P* = 0.0303 and Pearson r = 0.6267, *P* = 0.0292, respectively), and this correlation was similar between these mice (*P* = 0.8906 for the slope and *P* = 0.4332 for the intercept in the analysis of covariance). The effects of stress on active periods and body temperature dissipated before the end of the dark phase.

The hypothermic stressed mouse excluded from the above analyses showed a gradual, but large, decrease in body temperature, which was peaked at 5 h (ZT 17) after the stress (Fig. 1f). The body temperature at the peaked decrease was 31.1 °C and far below the 95% confidence interval of the linear regression of the other stressed mice as well as control mice. This severe hypothermia returned to the baseline before the social interaction test (ZT 13–15) on the next day (Fig. 1f).

### Social defeat stress disrupts positive valence of social sniffing in stress-susceptible mice

One day after the stress, we examined the effects of social defeat stress on social behaviors and exploratory activity in the social interaction test (Fig. 2a–d). Social defeat stress, but not cage transfer as the control, increased the time spent in the avoidance zone (Fig. 2b (left); *P* = 0.9997 in the control mice and *P* = 0.036 in the stressed mice for Sidak’s multiple comparisons tests: Fig. 2c (left); *P* = 0.0149 for Control vs Stress for Kolmogorov–Smirnov test). The stressed mice also spent less time in the interaction zone, though it was not statistically significant. Notably, the times in the avoidance zone and the interaction zone showed high individual variability, as previously reported^3,4^. The stressed mice that show social avoidance have been categorized as stress-susceptible mice, whereas those that do not show social avoidance as stress-resilient mice. In this study, we considered that the time in the avoidance or interaction zone was changed, if it exceeded the mean value of control mice by standard deviation. A subset of mice (6 out of 13 mice, 46.2%) showed significant increase in the avoidance zone and decrease in the interaction zone, so that these mice were categorized to stress-susceptible mice. By contrast, neither the time in the avoidance or interaction zone showed significant change in another subset of mice (7 out of 13 mice, 53.8%), thus stress-resilient mice. By definition, the distribution of the individual values was not overlapped between stress-susceptible mice and control mice or stress-resilient mice (Supplementary Fig. S1).

To quantify the level of social investigation, we manually counted the time of social sniffing to a target mouse of control and stressed mice, and found that social defeat stress significantly decreased the time of social sniffing (Fig. 2e). The sniffing time decreased in stress-susceptible mice (*P* = 0.0367 for Tukey multiple comparisons test) rather than stress-resilient mice (*P* = 0.126 for Tukey multiple comparisons test), suggesting that stress-induced decrease in the sniffing time stems from social avoidance in stress-susceptible mice. To further examine the time and patten of social sniffing, we employed deep learning-based detection of animal behaviors by DeepLabCut (Fig. 2f)^26^. We first confirmed that the sniffing time determined by DeepLabCut was highly correlated to that manually determined in all the experimental groups (Fig. 2g; Pearson r = 0.997, 0.9539 and 0.9927 as well as *P* < 0.0001 for control, stress and stress + sleep deprivation groups, respectively). Consistent with the manual detection of stress-induced decrease in the sniffing time, the stress decreased the number of sniffing bouts (Fig. 2h; 15.33 ± 3.703 and 8.385 ± 1.470 for control and stress groups, respectively; *P* = 0.0488 for unpaired *t* test). We examined the intensity of social sniffing measured by the duration of each sniffing bout, which has been used to analyze the motivation for social interaction^30^. Whereas social sniffing intensity was not significantly altered (Fig. 2i), it appeared to be positively correlated to the time spent in the interaction zone in control mice and stress-resilient mice, but not in stress-susceptible mice (Fig. 2j). Consistently, the positive correlation was statistically significant in the high-social-interest mice combined from control and stress-resilient mice (Pearson r = 0.7377, *P* = 0.0062), and its correlation coefficient was significantly different from that in stress-susceptible mice (*P* = 0.022 for ANCOVA). These findings show that control and stress-resilient mice exhibited positive valence of social sniffing associated with high social interest, which was disrupted in stress-susceptible mice.

### Stress-induced sleep-like inactivity contributes to multiple effects on social behaviors

Next, we tested the causal role for the stress-induced sleep-like inactivity on social behaviors by sleep deprivation for 6 h immediately after the stress (Fig. 1a). As expected, sleep deprivation blocked stress-induced sleep-like inactivity and body temperature decrease during the dark phase (Fig. 1b,d,f). In the social interaction test, the time in the interaction zone was significantly shorter in stressed mice with sleep deprivation (*P* = 0.0106 for Sidak’s multiple comparisons tests), but not in those without sleep deprivation (*P* = 0.1576 for Sidak’s multiple comparisons tests), than in control mice (Fig. 2b). Stressed mice with sleep deprivation (*P* = 0.0238 for Kolmogorov–Smirnov test), but not those without it (*P* = 0.2403 for Kolmogorov–Smirnov test), showed a distribution of the time spent in the interaction zone significantly different from that of control mice (Fig. 2c). Sleep deprivation also appears to decrease the proportion of stress-resilient mice, those with high social interest (2 out of 9 mice, 22.2%), as defined above. Indeed, the proportion of the high-social-interest mice was significantly smaller in stressed mice with sleep deprivation (*P* = 0.0406 for Fisher’s exact test), but not in those without sleep deprivation (*P* = 0.3331 for Fisher’s exact test), than in control mice (Fig. 2b). Consistent with the reduced social interest, sleep deprivation after the stress also decreased the social sniffing intensity (Fig. 2i), although neither the sniffing time nor the number of sniffing bouts were significantly affected (Fig. 2e,h). By contrast, sleep deprivation enhanced the exploratory activity in the presence of a target mouse (Fig. 2d; *P* < 0.0001 for Tukey’s test to compare the “Stress” and “Stress + Sleep dep” groups), with the positive correlation between the social sniffing intensity and the time in the interaction zone, thus the positive valence of social sniffing (Fig. 2j; Pearson r = 0.7669, *P* = 0.0159). Collectively, these findings show that sleep deprivation after the stress decreased social sniffing intensity along with reduced social interest, but enhanced the exploratory activity with the positive valence of social sniffing, suggesting multiple roles of stress-induced sleep-like inactivity for social behaviors.

### Social defeat stress activates sleep-related brain regions without affecting VLPO and MnPO

To examine neural mechanisms underlying stress-induced sleep-like inactivity, we examined c-Fos expression, a histological marker for neuronal activity, in brain regions related to sleep and wake regulations^13,31^ after either social defeat stress or cage transfer. The stress did not affect c-Fos expression in sleep-promoting brain regions, VLPO and MnPO (Fig. 3a,b). We previously reported that the stress did not significantly affect c-Fos expression in the locus coeruleus or dorsal raphe nucleus, which promote wakefulness^26^, relative to the control mice^27,32^. In this study, we found that the stress did not affect c-Fos expression in other wake-promoting brain regions, the medium septum, lateral hypothalamus, or laterodorsal tegmental nucleus, either. Thus, neither sleep-promoting nor wake-promoting brain regions showed neuronal activity consistent with stress-induced sleep-like inactivity. By contrast, the stress increased c-Fos expression in other sleep-related brain regions, such as the DMH and VLPAG, which regulate REM and non-REM sleep^15,16^, relative to the control mice (Fig. 3a,c,d). These findings show that social defeat stress activated sleep-related brain regions without affecting VLPO and MnPO in a manner consistent with the development of sleep-like inactivity.

## Discussion

Although stress induces sleep dysregulations, the role and mechanism remain elusive. In this study, we analyzed the social interest and motivation for social interaction by the proximity and social sniffing intensity to a target mouse, respectively, as well as the exploratory activity in the presence of a target mouse. We found that sleep deprivation after the stress decreased the motivation for social interaction along with reduced social interest. By contrast, the sleep deprivation enhanced the exploratory activity with the positive valence of social sniffing. We also found by c-Fos immunohistochemistry that the stress activated sleep-related brain regions, the DMH and VLPAG, without apparent effects on other sleep/wake-related brain regions. These findings show, for the first time, multiple effects of stress-induced sleep-like inactivity on social behaviors, and pave the way for elucidating sleep-related neural circuits that mediate these effects of stress.

Whereas previous studies reported that acute and chronic stress promotes sleep, most of these studies have not examined its behavioral roles. One recent study reported that sleep deprivation after social defeat stress exacerbated anxiety-like behaviors, suggesting the anxiolytic role of stress-induced sleep. In this study, we identified the role of stress-induced sleep in maintaining high social interest in stress-resilient mice associated with the positive valence of social sniffing. These findings collectively illustrate beneficial roles of stress-induced sleep. This study could analyze positive valence of social sniffing and its disruption by stress for the first time by the advent of deep learning-based detection of social sniffing. This deteriorating effect of stress was observed in stress-susceptible mice, but not in stress-resilient mice, suggesting that social defeat stress alters the behavioral outcome associated with social sniffing from social approach to social avoidance. Thus, the disruption of positive valence of social sniffing could be a novel behavioral readout of stress susceptibility. In addition, we identified novel effects of sleep deprivation in enhancing the exploratory activity in the presence of a social target with the positive valence of social sniffing. Stress-induced sleep could attenuate this exploratory behavior, thereby promoting negative appraisal of a social target and its association with social avoidance. Given that social defeat stress elicits associative learning between a social target and its negative outcome, stress-induced sleep could also promote the consolidation of this memory and consequently disrupt positive valence of social sniffing. Although sleep deprivation is a common method to analyze physiological and behavioral roles of sleep, this procedure not only perturbs sleep but also provides artificial stimuli including the exposure to novel objects and gentle handling. For example, the introduction of novel objects might be considered an environmental enrichment, even though it was only for 6 h, possibly providing an effect inverse to social defeat stress or other sources of uncertainty. Thus, it is important to manipulate specific neural mechanisms of stress-induced sleep-like inactivity once identified.

In this study, we found that social defeat stress did not activate sleep-promoting brain regions, the VLPO and MnPO, suggesting the lack of their involvement in stress-induced sleep. Combined with previous studies, we could not find the effect of stress in wake-promoting brain regions examined so far. However, since c-Fos immunostaining may not sensitively detect the decrease in neuronal activity, we cannot exclude possible involvement of these brain regions. The tuberomammillary nucleus and pedunculopontine tegmental nucleus that are known to promote wake also remain to be examined. Nonetheless, we found that social defeat stress activated the DMH and VLPAG that regulate REM/non-REM switching. Although whether DMH neurons can regulate total sleep is not reported, the activation of VLPAG neurons reduced wake periods along with increased non-REM sleep in a previous study^15^. Interestingly, these brain regions are activated by torpor-inducing stimuli^31^, such as fasting and cold exposure, and the DMH is crucial for optogenetically induced hibernation-like state^33^. Consistent with the activation of these brain regions, we found that social defeat stress decreases body temperature in a manner correlated to the proportion of active periods. Notably, one of the stressed mice showed severe hypothermia to 31.1 °C at its peak, which cannot be explained by reduced voluntary movements. Since this hypothermia was transient and returned to the baseline within a day, this hypothermia was not due to irreversible physical damages of the stressed mice or mechanical errors of the sensor. Thus, the activation of these brain regions could underlie stress-induced hypothermia. Whether these brain regions are involved in the effects of stress-induced sleep in social behaviors warrants future investigations.

Since depression and insomnia are more prevalent in females than in males, one of the limitations in this study is that we only analyzed male mice due to the technical constraint of repeated social defeat stress. The relationship between stress and sleep in female mice warrants future studies, using other stress models such as chronic mild stress as well as new designs of repeated social defeat stress applicable to female mice^34–37^. Nonetheless, our findings demonstrate multiple effects of stress-induced sleep on consequent social behaviors and highlight sleep-related brain regions associated with these effects. These effects of stress-induced sleep may be clinically relevant, since sleep deprivation is known to cause acute therapeutic effects for depression. However, the clinical use of sleep deprivation is limited so far, because it inevitably causes adverse outcomes as well. Neural mechanisms underlying the effects of stress-induced sleep need to be clarified to selectively augment beneficial arms of stress-induced sleep.

## Supplementary information

Supplementary Figure S1.
