# Supplementary material for: Stress-induced sleep-like inactivity modulates stress susceptibility in mice
Source: Sci Rep. 2020 Nov 13;10:19800. doi: 10.1038/s41598-020-76717-8 (PMC7666172; doi:10.1038/s41598-020-76717-8)
Supplement: Supplementary file 1 — Supplementary Figure S1. [file 41598_2020_76717_MOESM1_ESM.pdf]

Supplementary Information

Stress-induced sleep-like inactivity modulates stress susceptibility in mice

Midori Nagai[1,2], Hirotaka Nagai[1,2,*], Chisato Numa[1,2], Tomoyuki Furuyashiki[1,2,*]

[1]Division of Pharmacology, Graduate School of Medicine, Kobe University, Kobe, 650-0017, Japan

[2]Japan Agency for Medical Research and Development, Tokyo, 100-0004, Japan

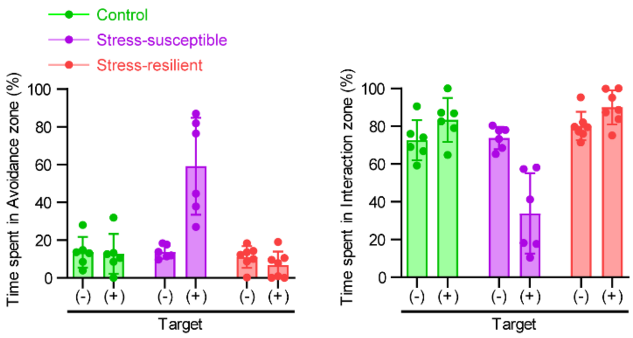

Supplementary Figure S1. Behavioral definitions of stress-susceptible and stress-resilient mice. The proportion of the time spent in the avoidance zone (left) and the interaction zone (right) are shown for control mice, stress-susceptible mice and stress-resilient mice during the habituation without an ICR target mouse (-) or the social interaction test with an ICR target mouse (+). Stress-susceptible and stress-resilient mice were categorized based on the proportion of the time spent in the avoidance and interaction zones (left and right, respectively). By definition, the distribution of the data points is not overlapped between stress-susceptible mice and control mice or stress-resilient mice. Values are expressed as means ± SD.
